# Critical roles for murine Reck in the regulation of vascular patterning and stabilization

**DOI:** 10.1038/srep17860

**Published:** 2015-12-11

**Authors:** Glícia Maria de Almeida, Mako Yamamoto, Yoko Morioka, Shuichiro Ogawa, Tomoko Matsuzaki, Makoto Noda

**Affiliations:** 1 Department of Molecular Oncology, Kyoto University Graduate School of Medicine, Yoshida-Konoe-cho, Sakyo-ku, Kyoto 606-8501, Japan

## Abstract

Extracellular matrix (ECM) is known to play several important roles in vascular development, although the molecular mechanisms behind these remain largely unknown. *RECK*, a tumor suppressor downregulated in a wide variety of cancers, encodes a membrane-anchored matrix-metalloproteinase-regulator. Mice lacking functional *Reck* die *in utero*, demonstrating its importance for mammalian embryogenesis; however, the underlying causes of mid-gestation lethality remain unclear. Using *Reck* conditional knockout mice, we have now demonstrated that the lack of *Reck* in vascular mural cells is largely responsible for mid-gestation lethality. Experiments using cultured aortic explants further revealed that Reck is essential for at least two events in sprouting angiogenesis; (1) correct association of mural and endothelial tip cells to the microvessels and (2) maintenance of fibronectin matrix surrounding the vessels. These findings demonstrate the importance of appropriate cell-cell interactions and ECM maintenance for angiogenesis and the involvement of Reck as a critical regulator of these events.

Correct vascular development is crucial for all aspects of tissue growth and physiology in vertebrates. In mammals, two families of cytokines; vascular endothelial growth factors (VEGFs) and angiopoietins, are known to play a lead role in angiogenesis^1^,^2^,^3^,^4^,^5^. Early events during sprouting angiogenesis involve specialization of activated endothelial cells into two distinct subtypes: namely, tip and stalk cells. VEGF stimulates the expression of tip cell markers, including Flk1 and Notch-ligands of which the Notch-ligands stimulate Notch-signaling in adjacent cells to suppress their tip cell phenotype (lateral inhibition) and induce the phenotype of lumen-forming stalk cells^6^. For vascular stabilization, endothelial tubes need to recruit, and be tightly associated with, mural cells (i. e., vascular smooth muscle cells and pericytes), whilst platelet-derived growth factor (PDGF) serves as a key attractant in this process^7^. This cell-cell interaction triggers the perivascular deposition of extracellular matrix (ECM) components, such as fibronectin (FN) and vascular basement membrane (vBM) to promote vessel maturation and stabilization^8^,^9^. Matrix metalloproteinases (MMPs) are also known to play major roles in the ECM-remodeling associated with angiogenesis^10^,^11^, although how this process is regulated remains to be elucidated.

*RECK*, conserved as a single gene from insects to primates, encodes a membrane-anchored regulator of multiple metalloproteinases, including several members of the MMP family^12^,^13^,^14^,^15^,^16^,^17^,^18^. In mammalian cells, *RECK* expression is downregulated by various external stimuli, such as growth factors, low cell density, and low oxygen^19^,^20^,^21^. *RECK* expression is also downregulated frequently in cancer cells, and restoration of RECK expression in such cells results in suppression of tumor angiogenesis, invasion, and metastasis in xenograft models^14^,^17^. Recent evidence indicates that several oncogenic microRNAs target *RECK* mRNA^20^,^22^,^23^,^24^,^25^,^26^, strengthening the notion that *RECK* is a tumor suppressor that is downregulated via various mechanisms during carcinogenesis.

Previous studies have also revealed the critical functions of *Reck* in mammalian development. Mice lacking *Reck*-expression die *in utero* around embryonic day 10.5 (E10.5), exhibiting reduced tissue integrity, arrested vasculogenesis^13^, and precocious neuronal differentiation^13^,^16^. A mouse mutant with reduced *Reck*-expression (*Reck*^*Low*/∆^, see below) demonstrates defects in limb patterning; a phenotype that can be explained by impaired Wnt7a-signalling due to tissue damage in limb-bud mesenchyme and overlaying dorsal epithelium^21^. In some rapidly proliferating tissues, such as embryos and uterine implantation chambers, *Reck* expression is abundant in both vascular endothelial cells and mural cells^27^. Dilated vessels with abnormal luminal shapes can be observed in these tissues in mice with reduced *Reck* expression. Abundant Reck-expression has also been found in fibroblastic cells associated with bifurcating vessels, leading to the speculation that Reck may play a role in non-sprouting angiogenesis (e.g., intussusception and pruning)^27^.

In the present study, we dissected the roles for *Reck* in different vascular cell types during angiogenesis by using multiple lines of newly developed *Reck* mutant mice. We also employed aortic ring assay (ARA)^28^,^29^ to assess the ability of aortic tissue explants to form small vessels (microvessels) *in vitro*. We found that selective inactivation of *Reck* in vascular mural cells caused embryonic death around E10.5 with vascular defects, suggesting that the mid-gestation lethality of *Reck*-null mice can be attributed to the absence of Reck in mural cells. In addition, we unexpectedly found that impaired Reck function leads to excessive sprouting of unstable microvessels *in vitro*, raising the possibility that the abnormal, dilated vessels found in *Reck*-deficient mice may arise by lateral fusion of unstable vessels rather than, or in addition to, abortive intussusception.

## Results

### Cell type-selective inactivation of *Reck in vivo*

**Figure 1:**
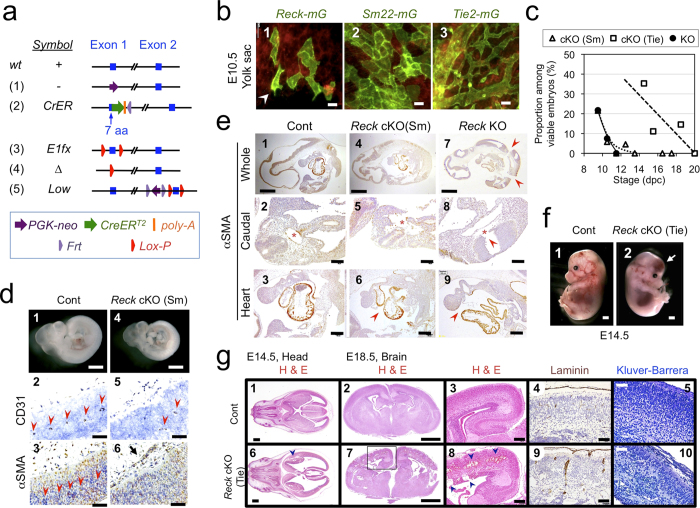
Cell type-selective inactivation of *Reck in vivo* using *Sm22-Cre* or *Tie2-Cre* mice. (**a**) *Reck* alleles used in this study. (**b**) Cells that expressed *Reck* (1), *Sm22* (2, mural cells), or *Tie2* (3, endothelial cells) emitted green fluorescence in the yolk sac of E10.5 embryos. For *Reck*, a pregnant mouse was injected with tamoxifen at 8.5 dpc. Arrowhead indicates a cell with ambiguous morphology. (**c**) Viability of global *Reck* KO mice (*Reck*^−/−^, filled circles with solid line) or tissue-selective *Reck* knockouts, *Reck* cKO (Sm) [*Reck*^*E1fx/∆*^; *Sm22-Cre*, triangles] or *Reck* cKO (Tie) [*Reck*^*E1fx/∆*^; *Tie2-Cre*, square]. Expected frequency was 25% in all cases. (**d**) Morphology of control (*Reck*^*E1fx/∆*^) and *Reck* cKO (Sm) mice at E10.5. Whole embryo (panels 1, 4) and the dorsal, peri-neural area of serial sagittal sections, immunostained for CD31 (panels 2, 5) or αSMA (panels 3, 6), are shown. Brown signals indicate immunoreactivity. Arrow indicates an abnormal peri-neural vessel. Arrowheads highlight CD31-positive small vessels within the neural tube. (**e**) Distribution of αSMA-immunoreactivity in the sagittal sections of control, *Reck* cKO (Sm), and *Reck* KO mice at E10.5. Whole section (top row), caudal area containing a cross-sectional view of the neural tube and dorsal aorta (second row), and the heart (third row) are shown. Arrowheads show broken sites in panels 7, 8 and missing pericardial membrane in panels 6, 9. Asterisks highlight dorsal aorta. (**f**) Gross morphology of the control (*Reck*^*E1fx/∆*^) and *Reck* cKO (Tie) mice at E14.5. Arrow indicates intra-cranial hemorrhage. (**g**) Brain morphology in sections of control (upper panels) or *Reck* cKO (Tie) mice (lower panels) at E14.5 (panels 1, 2, 6, 7) or E18.5 (3–5, 8–10) that were subjected to hematoxylin and eosin (panels 1–3, 6–8), anti-laminin (panels 4, 9), or Kluver-Barrera (panels 5, 10) staining. Arrowheads indicate intra-cranial hemorrhage. Scale bar: (**b**) 20 μm; (**d**) 1 mm (1, 4), 50 μm (other panels); (**e**) 1 mm (1, 4, 7), 100 μm (2, 5, 8), 200 μm (3, 6, 9); (**f**) 1 mm; (**g**) 1 mm (1, 2, 6, 7), 200 μm (3, 8), 100 μm (4, 9), 50 μm (5, 10).

The engineered *Reck* alleles in mice used in this study are listed in Fig. 1a: (1) *Reck*^*-*^, the original null-allele^13^, (2) *Reck*^*CrER*^, expressing the tamoxifen-regulatable Cre recombinase CreER^T2^ from the *Reck* locus (Matsuzaki et al. in preparation); (3) *Reck*^*E1fx*^, containing two lox-P sites flanking exon-1; (4) *Reck*^*∆*^, a novel null-allele lacking proximal promoter plus exon-1; and (5) *Reck*^*Low*^, a hypomorphic allele expressing *Reck* at ~50% of the wild type (*wt*) level^21^. In addition, we utilized two Cre transgenic lines, *Sm22-Cre*^30^ and *Tie2-Cre*^31^, to induce *loxP*-recombination selectively in mural and endothelial cells, respectively.

**Figure 2:**
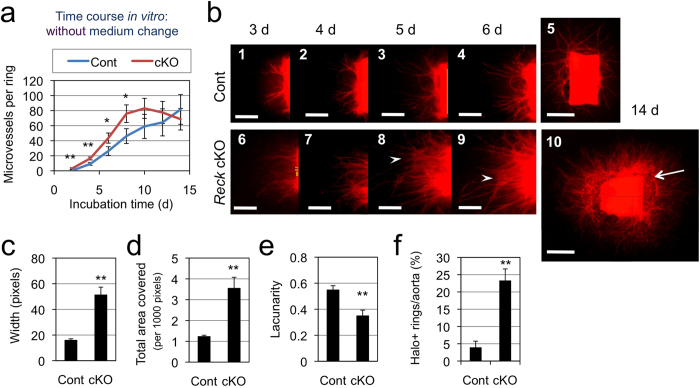
Effects of *Reck*-deficiency on microvessel formation in ARA. (**a**) Time course of microvessel formation (number per ring). Aortic rings from 5-weeks old control (*Reck*^*+/CrER*^; *mTmG*, blue line, n = 9) or *Reck* cKO (*Reck*^*E1fx/CrER*^; *mTmG*, red line, n = 4) mice were subjected to ARA (no medium change), and the number of microvessels was counted every other day from day 2 to 14. (**b**) Fluorescent micrographs of a control (panels 1–5) or *Reck* cKO (panels 6–10) ring after incubation for indicated period of time in ARA without medium changes. Arrow indicates peri-aortic halo, and arrowheads highlight aggregating microvessels. Scale bar: 1 mm (5, 10), 0.5 mm (other panels). (**c**) Relative width of microvessels measured using ImageJ on high magnification images. Summary of 6 independent experiments (n = 36 rings for Cont and 30 rings for *Reck* cKO in total). (**d, e**) Parameters measured using AngioTool^44^. Summary of 8 independent experiments for Cont (46 rings) and 7 experiments for *Reck* cKO (43 rings). (**f**) Frequency of rings exhibiting peri-aortic halo. Summary of 6 independent experiments for Cont (78 rings) and 3 independent experiments for *Reck* cKO (30 rings).

**Figure 3:**
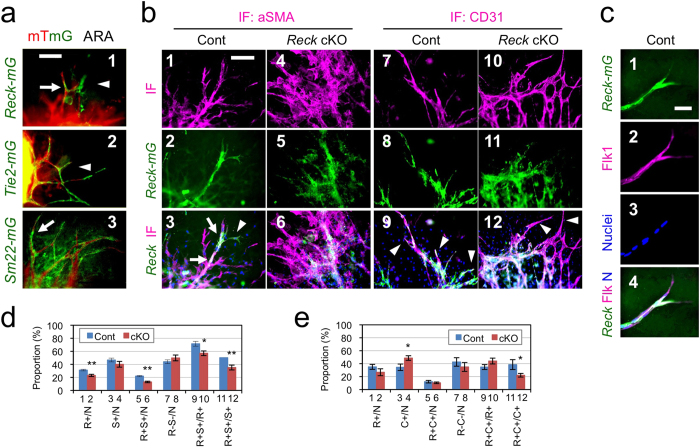
Identity and behavior of *Reck-mG* cells in ARA. (**a**) Morphology and microvessel-association of *Reck-mG* (1), *Tie2-mG* (2), and *Sm22-mG* (3) cells (green) in ARA at day 8. (**b**) Effects of *Reck*-deficiency on the nature of, and relationship between, *Reck-mG*, endothelial, and mural cells. Aortic rings from the control (*Reck*^*+*/*CrER*^; *mTmG*) or *Reck* cKO (*Reck*^*E1fx/CrER*^; *mTmG*) mice were subjected to ARA and immunostained for αSMA (magenta in panels 1, 3, 4, 6) or CD31 (magenta in panels 7, 9, 10, 12) at day 11. Green signals represent *Reck-mG* cells*. Reck-mG*/αSMA double positive staining could be observed at microvessel stalks from control mice (white stain, arrow, panel 3). In control cultures, *Reck-mG*/CD31 double positive staining could be observed at microvessel tips (white stain, arrowheads, panel 9) and *Reck-mG* single positive staining found at microvessel stalks (green stain, panel 9). This was lost in *Reck* cKO mice as observed by the lack of *Reck-mG*/CD31 double positive at the microvessel tips (arrowheads, panel 12). (**c**) Flk1 immunoreactivity in a control microvessel. The control culture as in (**b**) was immunostained for a vascular tip-cell marker, Flk1/Vegfr2 (magenta in panel 2 and 4). (**d**) Frequency of various cells in *Reck-mG*/αSMA double labeling experiments as shown in (**b**), panels 1–6. (**e**)Frequency of various cells in *Reck-mG*/CD31 double labeling experiments as shown in (**b**), panels 7–12. N = total nuclei, R = *Reck-mG*, S = αSMA, C=CD31, “+” means “-positive”. Number of images analyzed: (**d**) Cont (n = 17), *Reck* cKO (n = 12). (**e**) Cont (n = 6), *Reck* cKO (n = 9). Bars 1–8 represent proportion among all cells. Bars 9 and10 represent proportion among *Reck-mG* cells. Bars 11 and 12 represent proportion among αSMA-positive cells (**d**) or CD31-positive cells (**e**). Aortic rings were prepared from at least 3 animals per group. Scale bar: (**a**, **b**) 100 μm, (**c**) 20 μm.

Visualization of *Reck*-positive cells in the yolk sac at embryonic day 10.5 (E10.5), using mice carrying the *Reck*^*CrER*^ allele together with the *mTmG* reporter system^32^, revealed that the majority of *Reck-mG* cells (green; Fig. 1b-1) exhibit cobble stone-like alignment resembling that of *Sm22-mG* vascular mural cells (green, Fig. 1b-2). This phenotype was widespread, rather than the dendritic pattern of *Tie2-mG* vascular endothelial cells (green, Fig. 1b-3), although some cells with ambiguous morphology were also observed (e.g, Fig. 1b-1, arrowhead).

**Figure 4:**
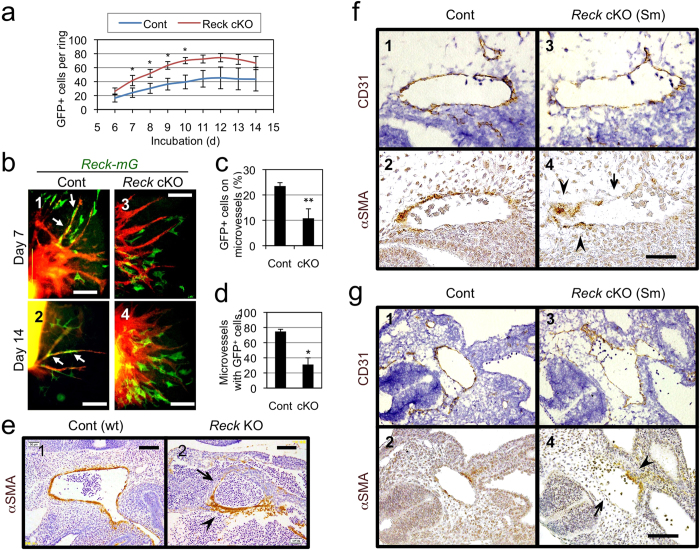
Effects of *Reck* on vessel-association of *Reck-mG* cells in ARA and mural cells *in vivo*. (**a**) Time course for the number of *Reck-mG* cells in ARA. Aortic rings from the 5-weeks-old control (n = 10) or *Reck* cKO (n = 7) mice were subjected to ARA and all *Reck-mG* cells surrounding each ring (irrespective or their association with microvessels) were counted from day 6 to 14. (**b**) Distribution and morphology of *Reck-mG* cells (green) at day 7 and 14 in ARA. In control cultures, *Reck-mG* cells are tightly associated with microvessel stalks (arrows in panel 1 and 2) but this is impaired in *Reck* cKO (panel 3 and 4). (**c**) Frequency of *Reck-mG* cells tightly associated with microvessels at day 8. n = 3. (**d**) Frequency of microvessels with tightly associated *Reck-mG* cells at day 8. n = 3. (**e–g**) Effect of *Reck*-deficiency on the distribution of mural cells *in vivo*. (**e**) Sagittal section of wild type (Cont) or *Reck*^−/−^ (*Reck* KO) mouse embryos at E10.5 immunostained for αSMA (brown). Micrographs focusing on large aorta are shown. (**f,g**) Sagittal sections of *Reck*^*E1fx/∆*^ mice (control; panels 1, 2) and *Reck* cKO (Sm) mice (panels 3, 4) at E10.5. Adjacent sections were immunostained for CD31 (with Hematoxylin counter-stain; panels 1, 3) and for αSMA (panels 2, 4), respectively. Micrographs focusing on large aortas in the middle (**f**) and caudal (**g**) part of the body are shown. In (**e**–**g**), arrowhead indicates an area of vessel wall abundant in mural cells, and arrow highlights an area where the mural cell layer is very thin or absent. Scale bar: (**b**) 200 μm (panel 2) and 50 μm (other panels), (**e**) 100 μm, (**f**) 50 μm, (**g**) 100 μm.

**Figure 5:**
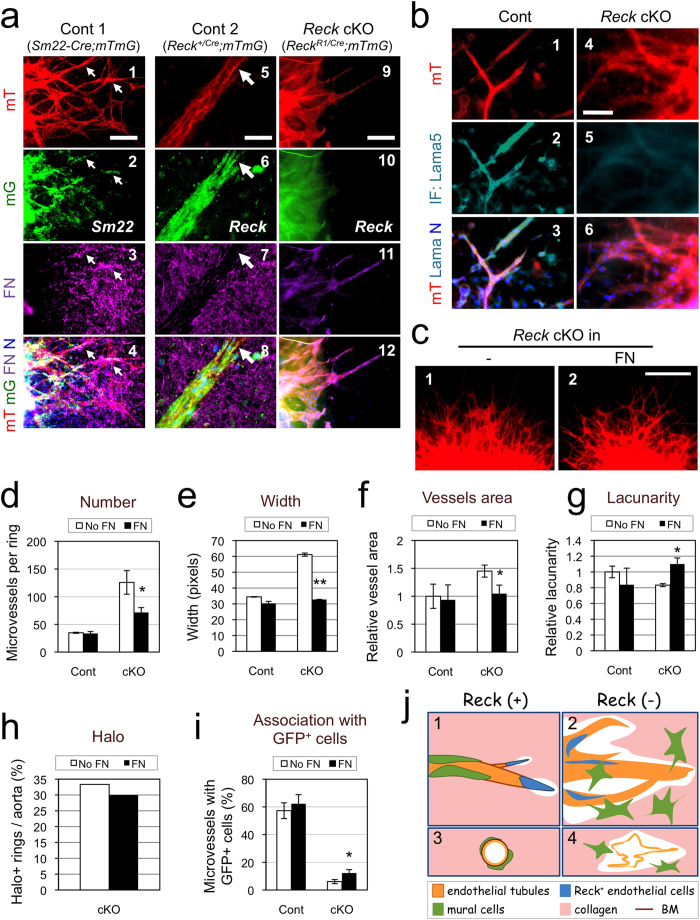
Functional relationship between Reck and FN in microvessel formation in ARA. (**a**) Effects of *Reck*-deficiency on FN fibrils in ARA. Aortic rings from control 1 (*Reck*^*+*/*+*^; *Sm22-Cre*; *mTmG*), control 2 (*Reck*^*+*/*CrER*^; *mTmG*), and *Reck* cKO (*Reck*^*E1fx/CrER*^; *mTmG*) mice pretreated with tamoxifen were subjected to ARA and stained for FN (magenta, panels 3, 4, 7, 8, 11, 12) at day 10. Note that in the control samples, FN (magenta) signals are abundant around the extending sprouts (panels 3, 7), particularly accumulated in the areas where mT (red) and Sm22 (green) signals overlap (arrows in panels 1–4), but sparse near the tip of the sprouts (arrow in panels 5–8). Paucity of FN (magenta) signals in *Reck* cKO sample (panels 9–12) could also be observed. (**b**) Similar cultures stained for laminin α 5 (Lama5, turquoise). (**c**–**i**) Effects of exogenous FN on the phenotype of *Reck* cKO microvessels in ARA. ARAs were performed without or with addition of FN (1 μg/ml) in the gel. (**c**) Typical morphology of microvessels from *Reck* cKO aortic rings after incubation for 9 days in the absence (1) or presence (2) of exogenous FN. (**d–i**) Indicated parameters were measured as described in Fig. 2 **a, c-f** (**d–h**) and Fig. 4d (**i**) using micrographs (as shown in **c**) taken at day 10. Cont: n ≥ 14 rings (3 aortae); *Reck* cKO: n ≥ 43 rings (6 aortae). (**j**) A model consistent with our findings *in vitro*. When Reck is present (panels 1, 3), endothelial sprouting led by tip cells (blue) is appropriately regulated; tight association of mural cells (green) with endothelial tubules (orange) is promoted; and individual vessels are stabilised. When Reck is absent or reduced (panels 2, 4), endothelial sprouting and collagenolysis are activated; tip cells and mural cells localise inappropriately; and microvessels destabilise, permitting lateral fusion and ectopic anastomoses. Scale bar: (**a**) 20 μm in panels 5–8, 100 μm in others; (**b**) 50 μm; (**c**) 1 mm.

First, to assess the contribution of mural *Reck* to vascular development, *Reck* was inactivated using the *Sm22-Cre* driver mouse (Supplementary Fig. S1a,b). The resulting mutant mice, termed *Reck* cKO (Sm), were reminiscent of *Reck*^−/−^ mice^13^,^27^ in that they died around E10.5 (Fig. 1c, triangles) with smaller body size (Fig. 1d-4), poor vascularization in the neural tube (Fig. 1d-5), and dilated perineural vasculature with peculiar luminal shape (Fig. 1d-6). As reported previously, *Reck*^−/−^ embryos displayed reduced tissue integrity (Fig. 1e-7) with frequent breakage of the neural tube (e.g., Fig. 1e-8, arrowhead)^2^. Although *Reck* cKO (Sm) embryos were not as fragile (Fig. 1e-4), they shared some phenotypes with *Reck*^−/−^ mice such as frequent breakage of dorsal aorta (Fig. 1e-5) and pericardial membrane (Fig. 1e-6). Hence, the mid-gestation lethality of *Reck*-null mice^13^ may largely be attributable to the *Reck*-deficiency in mural cells.

Next, to assess the contribution of endothelial *Reck*, *Reck* was inactivated using the *Tie2-Cre* mouse (Supplementary Fig. S1c,d). Although the mutant mice, termed *Reck* cKO (Tie), survived beyond E10.5, they died before birth (Fig. 1c, squares) and exhibited intra-cranial hemorrhage (Fig. 1f-2; Fig. 1g, panels 6–8) with severe abnormalities in vasculature and cytoarchitecture in their cerebral cortex (Fig. 1g, panels 7–10). Hence, *Reck* in Tie2-positive cells is indispensable for later-stage embryogenesis, particularly within the brain.

### Effects of *Reck*-deficiency on microvessel formation

To understand the roles of *Reck* at the cellular level, ARAs^28^,^29^ were utilized, which allowed assessment of the ability of dorsal aorta tissue pieces (aortic rings) to form microvessels *in vitro*. Aortae from 5-weeks old, tamoxifen-induced *Reck* knockout (*Reck* cKO) and control (Cont) mice carrying the *mTmG* reporter were used. Under optimized conditions (Supplementary Figs S2 and S3a), control aortic rings showed a slow but steady increase in microvessel number over the time course observed (Fig. 2a, blue line), whereas *Reck* cKO samples showed an initial rapid increase (up to day 10) followed by a decline (from day 12) in the number of microvessels (Fig. 2a, red line). This decline was accompanied by aggregation and thickening of microvessels (Fig. 2b-8, 9, arrowheads; Supplementary Fig. S3b,c). Morphometry of fluorescent images (Fig. 2c-f and Supplementary Fig. S4) indicated that the *Reck* cKO microvessels were wider (Fig. 2c) and covered a broader area (Fig. 2d) with lower lacunarity (Fig. 2e). In addition, *Reck* cKO aortic rings were often accompanied by peri-aortic halos, indicating increased local lysis of collagen gel^33^ (Fig. 2b-10, arrow; Fig. 2f). Thus, *Reck*–deficiency leads to the formation of an excessive number of unstable microvessels in this assay.

### Identity of *Reck-mG* cells

To determine how *Reck*-deficiency leads to such microvessel phenotypes, the nature of *Reck-mG* cells in ARA was examined. In control cultures, some *Reck-mG* cells localize at microvessel tips (Fig. 3a-1, arrowhead), whilst others are associated with microvessel stalks (Fig. 3a-1, arrow). The former look similar to the *Tie2-mG* or CD31-positive endothelial cells (Fig. 3a-2, arrowhead; Supplementary Fig. S5a, magenta) and the latter the *Sm22-mG*-positive mural cells (Fig. 3a-3, arrow; Supplementary Fig. S5a, green). Immunofluorescent staining for another mural marker, αSMA, often detected *Reck-mG*/αSMA double-positive (i.e., mural *Reck-mG*) cells surrounding microvessel stalks (Fig. 3b-3 and Supplementary Fig. S6-3, white signals as indicated by arrows); CD31-staining detected *Reck-mG*/CD31 double-positive (endothelial *Reck-mG*) cells near microvessel tips (Fig. 3b-9 and Supplementary Fig. S6-9, white signals as indicated by arrowheads) along with *Reck-mG* single-positive (non-endothelial *Reck-mG*) cells surrounding microvessel stalks (Fig. 3b-9 and Supplementary Fig. S6-9, green signals). *Reck-mG* cells found near microvessel tips were often positive for a vascular tip cell marker, Flk1 (Fig. 3c-2, magenta). These results, together with morphometric data (Fig. 3d, e), indicate that *Reck-mG* cells in control cultures contain both mural (ca. 70%; Fig. 3d, bar 9) and endothelial (ca. 30%; Fig. 3e, bar 9) populations.

### Effects of *Reck* on the number and behaviors of *Reck-mG* cells

*Reck*-deficiency had multiple effects on *Reck-mG* cells. First, the number of *Reck-mG* cells surrounding an aortic ring was increased (Fig. 4a) whilst Ki67-staining revealed increased proliferation of both microvessel-associated and non-associated cells (Supplementary Fig. S5b and c, magenta). Second, the proportion of mural *Reck-mG* cells was decreased (Fig. 3d, bars 6, 12) whereas that of *Reck-mG*-negative endothelial cells was increased (Fig. 3e, bar 4). Third, endothelial *Reck-mG* cells were seldom found at microvessel tips in *Reck* cKO samples (Fig. 3b-12 and Supplementary Fig. S6-12, arrowheads; Supplementary Fig. S5d). Fourth, tight association of *Reck-mG* cells with microvessel stalks was somehow impaired (Figs. 4b-3 and -4; Fig. 4c, d); consistent to this finding *in vitro*, large aortae were frequently surrounded partially and unevenly by αSMA-positive mural cells that are not so tightly associated with a vessel as the control (Figs 4e-1) in sections of *Reck*^−/−^ embryos at E10.5 (Figs 4e-2). Partial and uneven coverage of large aortae by αSMA-positive cells was also found in *Reck* cKO (Sm) embryos at E10.5 (Fig. 4f, g), suggesting a cell-autonomous function of Reck manifested in this phenotype. In addition, the irregularly spaced small vessels (CD31-positive) in the neural tube of *Reck* cKO (Sm) mice were seldom accompanied by αSMA-positive mural cells (Figs 1d-5 and 6). Taken together, these findings suggest that *Reck* is required to achieve adequate compositions of, and interactions between, vessel-forming cells.

### Functional relationship between Reck and FN in microvessel formation

FN and its receptor are known to be protected by RECK^18^,^34^,^35^. In ARA with *Reck*-positive cells, abundant FN fibrils could be visualized by immunofluorescent staining (Fig. 5a, panel 3 and 7), and prominent signals were found in the areas where mural (*Sm22-mG*) cells tightly associated with microvessels (Fig. 5a, panels 1–4, arrows). Near the tips of these microvessels, localized loss of FN fibrils were found near microvessel tips (Fig. 5a, panel 5–8, arrow). In *Reck* cKO cultures, signals for both FN (Fig. 5a-11) and laminin α 5 (Lama5), a component of vBM (Fig. 5b-5), were dampened and diffuse. When *Reck* cKO aortic rings were embedded in collagen gel supplemented with purified FN (Fig. 5c), some *Reck* cKO phenotypes, such as increased number, width, and aggregation of microvessels, increased area covered by microvessels, and decreased lacunarity of vascular network, were significantly suppressed (Fig. 5c-g) with increased perivascular FN- and Lama5-immunoreactivity (Supplementary Fig. S7a and b). Other *Reck* cKO phenotypes, such as peri-aortic halo and poor association of *Reck-mG* cells with microvessels, were not fully suppressed by FN supplementation (Fig. 5h, i). Hence, some *Reck* cKO phenotypes in ARA, such as excessive sprouting and destabilized microvessels, may be attributable to the reduced ambient FN in the absence of *Reck*.

## Discussion

In this study, the importance of Reck in both mural and endothelial cells was documented *in vivo* and *in vitro*. Although selective inactivation of *Reck* in mural cells *in vivo* resulted in mid-gestation lethality and vascular defects reminiscent of *Reck*-null mice (Fig. 1c–e and ref. 2)*Reck*-inactivation in Tie2-positive cells also resulted in embryonic death, albeit at later stages with major impacts in the brain (Fig. 1c, f,g). *In vitro*, *Reck*-positive cells contribute to both mural and endothelial lineages (Fig. 3) whilst *Reck*-inactivation results in increased sprouting, decreased microvessel stability (Fig. 2) and altered composition (Fig. 3d–f) alongside a change in behavior that includes defective localization and reduced association (Figs 3b,4; Supplementary Fig. S5d) of vascular cells whose normal counterparts express *Reck*.

Senger, Stratman, and Davis have proposed that mural-endothelial interaction triggers perivascular FN-deposition and subsequent vBM-deposition required for vascular stability *in vivo* 8,9. Our data *in vitro* suggest that Reck plays a key role in this process by protecting FN from degradation (Supplementary Fig. S7c). The failure of FN to fully normalize the association of *Reck-mG* cells to the microvessels (Fig. 5i) fits with this model, (Supplementary Fig.S7c) which places the mural-endothelial interaction upstream of FN-deposition; raising a new question as to how Reck promotes this upstream event. Attenuated PDGF-receptor immunoreactivity found in *Reck*-deficient cells (Supplementary Fig. S8a and b) may be suggestive of its involvement in this failure, since PDGF is known to play a key role in pericyte-recruitment by endothelial cells 36 (Supplementary Fig. S8d-1).

The mechanism underlying the contribution of *Reck*-deficiency to the mislocalization of *Reck-mG* tip cells is presently unclear but several hypotheses can be envisaged. In *Reck* cKO culture, Flk1 signals were dampened and scarce in cells at the microvessel tips (Supplementary Fig. S5d, magenta), suggesting abortive tip-stalk specification. *Reck*-deficient mice exhibit precocious neuronal differentiation, and this has been explained by attenuated Notch-signaling in neural precursor cells, due to de-regulated Adam10 that clips off Notch-ligands from adjacent cells^16^. In the vascular system, Notch signaling is known to suppress the tip cell phenotype and to promote acquisition of the stalk cell phenotype^6^. Hence, an obvious model is that attenuated Notch-signaling in this system leads to ectopic expression of tip cell phenotype, which can explain the excessive sprouting found in *Reck* cKO rings in ARA. This model, however, predicts upregulation and/or ectopic expression of tip-cell markers (such as Flk1), but this was not the observed (Supplementary Fig. S5d). An alternative model involves direct Flk1-downregulation, for instance, by proteolytic cleavage that may scramble tip-stalk specification (Supplementary Fig. S8d-2). Another potential model is binding between VEGF and FN^37^ that somehow contributes to tip-stalk association or specification (Supplementary Fig. S8d-3), which fits with our finding that FN may suppress the mislocalization of tip cells found in *Reck* cKO samples to some extent (Supplementary Fig. S8c; Fig. 5i).

Likewise, multiple models can be proposed to explain the increased cell proliferation found in *Reck* cKO cultures. Since Notch-signaling is known to confer quiescence to the vessels^6^, reduced Notch-ligands may promote cell proliferation. Alternatively, as observed in colon cancer cells^38^, *Reck*-deficiency may promote cell cycle progression by upregulating Skp2, thereby downregulating the Cdk inhibitor p27. These issues need to be addressed in future studies.

We have previously speculated that dilated vessels found in *Reck*-deficient mice may result from impaired non-sprouting angiogenesis (i.e., intussusception)^27^. The present data, however, support an alternative model that dilated vessels could result from aggregation and lateral fusion of excessive, unstable microvessels (Fig. 5j-4). Our data also raise the interesting possibility that Reck may serve as a key regulator of vascular morphogenesis by regulating the number and size of the vessels, the area covered by the vessels, as well as the site and timing of anastomoses (Fig. 5j). We also speculate that RECK-dysfunction may underlie various conditions that give rise to fragile, leaky blood vessels, such as cancers^39^ and RASopathies^40^, since several activated oncogenes, including mutated RAS, strongly suppress *RECK* expression^12^,^41^. Compounds capable of upregulating endogenous *RECK*^42^ may be useful in ameliorating such conditions.

## Methods

### Mice

Animal experiments were approved by Animal Experimentation Committee, Kyoto University and conducted in accordance with its regulation. Generation and genotyping of mice carrying the *Reck*^*CrER*^ (also known as *Reck-CreER*^*T2*^ or *KI*), *Reck*^*E1fx*^ (also known as *R1*), *Reck*^*∆*^, or *Reck*^*Low*^ (also known as *R2neo*) allele has been described elsewhere^21^. To evaluate the roles of *Reck* in mural cells, *Reck*^*+/∆*^ mice were crossed with mice carrying *Sm22-Cre* transgene^30^ to obtain *Reck*^*+/∆*^; *Sm22-Cre* male mice, which were then crossed with *Reck*^*E1fx/E1fx*^ female mice, and their pups harvested at various stages for genotyping and morphological studies. To evaluate the roles of *Reck* in endothelial cells, *Reck*^*+/∆*^ mice were crossed with *Tie2-Cre* transgenic mice^31^ to obtain *Reck*^*+/∆*^; *Tie2-Cre* male mice, which were crossed with *Reck*^*E1fx/E1fx*^ female mice, and their pups examined at various stages. To visualise cells expressing *Tie2*, *Sm22*, or *Reck*, female mice homozygous for the allele containing the *mTmG* transgene^32^ were crossed with male mice carrying the *Tie2-Cre*, *Sm22-Cre*, or *Reck*^*CrER*^ transgene to obtain pups carrying both the *mTmG* and Cre transgenes. To test the roles of *Reck* in microvessel formation *in vitro*, *Reck*^*+/CrER*^; *mTmG*/*mTmG* male mice were crossed with *Reck*^*E1fx/E1fx*^ female mice (both in ICR background), and *Reck*^*E1fx/CrER*^; *mTmG* mice (*Reck* cKO) were selected. To generate a matched control, *Reck*^*+/CrER*^ male mice were crossed with *mTmG*/*mTmG* female mice, and *Reck*^*+/CrER*^; *mTmG* mice (Cont) were selected. Mice carrying the *Reck*^*CrER*^ transgene were treated four times by daily intra-peritoneal injections of tamoxifen (80 mg/kg), and then two days later, aortae were harvested for ARA.

### Histology

Mouse embryos were fixed, sliced (10 μm-thick), and stained with Hematoxylin and Eosin^21^, by immunohistochemistry with anti-CD31, anti-αSMA, or anti-laminin^27^, or by Kluver-Barrera method^43^ as described previously. Tissues from mice carrying the mTmG reporter were fixed, incubated overnight in 30% sucrose, embedded in O.C.T, frozen at –80 oC, sliced (5μm-thick), and observed with a fluorescent microscope.

### Aortic ring assay

ARA was performed following the protocol of Baker *et al.*^29^ with some modifications. The following conditions, optimised as shown in Supplementary Fig. S2, were used unless otherwise stated. Thoracic aortae were dissected from 5-week old transgenic mice after overnight starvation. The peri-aortic fibroadipose tissue and blood were carefully removed with fine microdissecting forceps, and aorta tunics were preserved without damage. After cleaning, aortae were cut into 1 mm-thick rings, rinsed 3 times with PBS (−), and incubated overnight in Opti-MEM at 37 °C. The rings were then embedded in a mound of gel prepared on ice by mixing 5 volumes of 3 mg/ml collagen type I-A (Nitta Gelatin, Osaka, Japan), 3 volumes of 3 mg/ml collagen type IV (Nitta Gelatin, Osaka, Japan), 1 volume of 10× DMEM, and 1 volume of reconstitution buffer (2.2 g NaHCO_3_ in 100 ml of 0.05 N NaOH and 200 mM HEPES). For embedding, 50 μl of the collagen mixture was placed into wells of 96-well plates and incubated at 37 °C for 10 minutes to form a basal gel. Aortic rings were placed on the basal gel and covered with 10 μl of cold collagen mixture. After 20-min incubation at 37 °C, each well was fed with 200 μl Opti-MEM (Invitrogen) supplemented with 20 ng/ml VEGFa (R&D systems), 5% fetal horse serum (Cell Culture Laboratories, Ohio, USA), 100 U/ml penicillin and 100 μg/ml streptomycin. The dishes were tightly sealed with parafilm, kept at 37 °C for two weeks, and examined every day under an inverted microscope. Micrographs were taken every day up to day 14 for morphometric analyses. In some experiments (Fig. 5c–i; Supplementary Fig. S7, S8c), bovine plasma fibronectin (Sigma, F1141) was added to the gel at 1 μg/ml.

### Morphometry

Microscopic images were recorded with a digital camera at various time points and magnification, depending on the properties to be assessed: x40 on day 3 to 7 for microvessel growth (number and length), x100 on day 8 and 9 for microvessel width, and x200 on day 10 to 14 for the cells associated with microvessels. The images were analysed using ImageJ to determine the length, width, and anastomosis of microvessels and the area covered by them. The numbers of microvessels were counted manually, following the criteria described by Aplin *et al.*^33^, where outgrowth constituted by singular cells such as fibroblasts, pericytes, and macrophages or segmented sprout structures were not included. AngioTool^44^ was also employed to quantify microvessel area, branching points (total junctions), total length, end points, and lacunarity.

### Immunofluorescent staining

Aortic rings extending microvessels were fixed with 4% formalin, permeabilised with 0.25% Triton-X for 15 min and incubated in PBS (+) containing 10% goat serum for 1 h at room temperature to block non-specific binding.After being rinsed with PBS (+) for 5 min they were then incubated overnight with primary antibody diluted in PBLEC^29^. After rinsing with PBS (+) (3 × 10 min), samples were then incubated for 30 min at 37 °C in a cocktail of Alexa Fluor (488, 555, or Cy5) conjugated with appropriate secondary antibodies in PBLEC. Samples were rinsed then incubated (1 min) with Hoechst33342 and mounted using Fluoromount (Diagnostic BioSystems). Images were recorded using a fluorescence microscope (OlympusX70 or Keyence BZ-9000). The following primary antibodies were used: laminin (Progen 10765), CD31 (BD Pharmingen 550274), Reck [RECK-F, polyclonal rabbit antibodies (Matsuzaki et al., in preparation) and 5B11D12^12^]; α-Sma (DAKO MO85), fibronectin (BD Biosciences, 610078), PDGFR-beta (Santa Cruz Biotechnology sc-432), Flk-1 (Santa Cruz Biotechnology sc-625), LAMA5 (Sigma-Aldrich SAB4501720) and Ki67 (Leica Biosystems NCL-Ki67p).

### Statistics

Quantitative data are presented in the form of mean ± sem in graphs. Significance of difference between two sets of data were assessed by Student’s t-test using the TTEST function in Excel (Microsoft), and the results are presented by symbols: *P < 0.05, **P < 0.01.

## Additional Information

**How to cite this article**: Almeida, G. M. *et al.* Critical roles for murine Reck in the regulation of vascular patterning and stabilization. *Sci. Rep.* **5**, 17860; doi: 10.1038/srep17860 (2015).

## Supplementary Material

Supplementary Information
