# Corrigendum: Critical roles for murine Reck in the regulation of vascular patterning and stabilization

**DOI:** 10.1038/srep20749

**Published:** 2016-03-04

**Authors:** Glícia Maria de Almeida, Mako Yamamoto, Yoko Morioka, Shuichiro Ogawa, Tomoko Matsuzaki, Makoto Noda

Scientific Reports 5: Article number: 1786010.1038/srep17860; published online: 12112015; updated: 03042016

In this Article, an additional affiliation for Glícia Maria de Almeida was omitted. The correct affiliation is listed below:

Graduate School of Biostudies, Kyoto University, Yoshida-Konoe-cho, Sakyo-ku, Kyoto 606-8501, Japan

