# Supplementary material for: Critical roles for murine Reck in the regulation of vascular patterning and stabilization
Source: Sci Rep. 2015 Dec 11;5:17860. doi: 10.1038/srep17860 (PMC4675993; doi:10.1038/srep17860)
Supplement: Supplementary Information [file srep17860-s1.doc]

Critical roles for murine Reck in the regulation of vascular patterning and stabilization

Glícia Maria de Almeida, Mako Yamamoto, Yoko Morioka, Shuichiro Ogawa, Tomoko Matsuzaki & Makoto Noda

Supplementary Figures

**
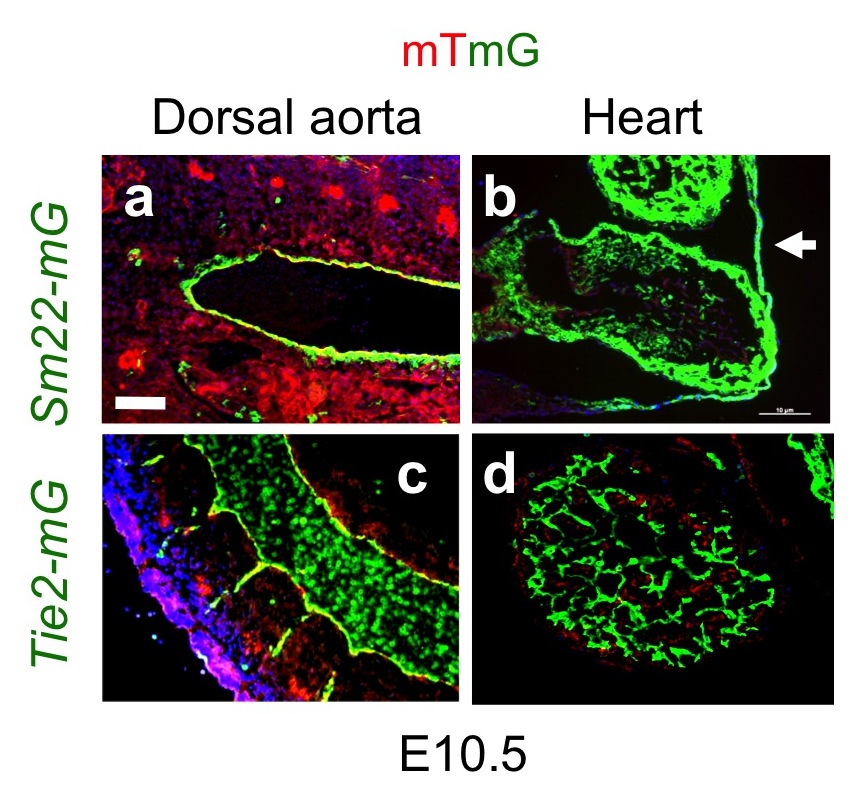
**

**Figure S1 | Cell type selectivity of the vascular Cre-driver mice used in this study.** *Sm22-mG* (a, b) and *Tie2-mG* (c, d) cells (green) in the dorsal aorta (a, c) and heart (b, d) in E10.5 embryos. Red and blue signals represent untagged cells and cell nuclei, respectively. Arrow indicates pericardial membrane. Scale bar: 100 µm. *Tie2-mG* cells are found in hematopoietic cells as well as vascular endothelial cells at this stage (c); Tie2 is known as a hemangioblast marker which is also expressed in early hematopietic progenitor cells^1^.

**
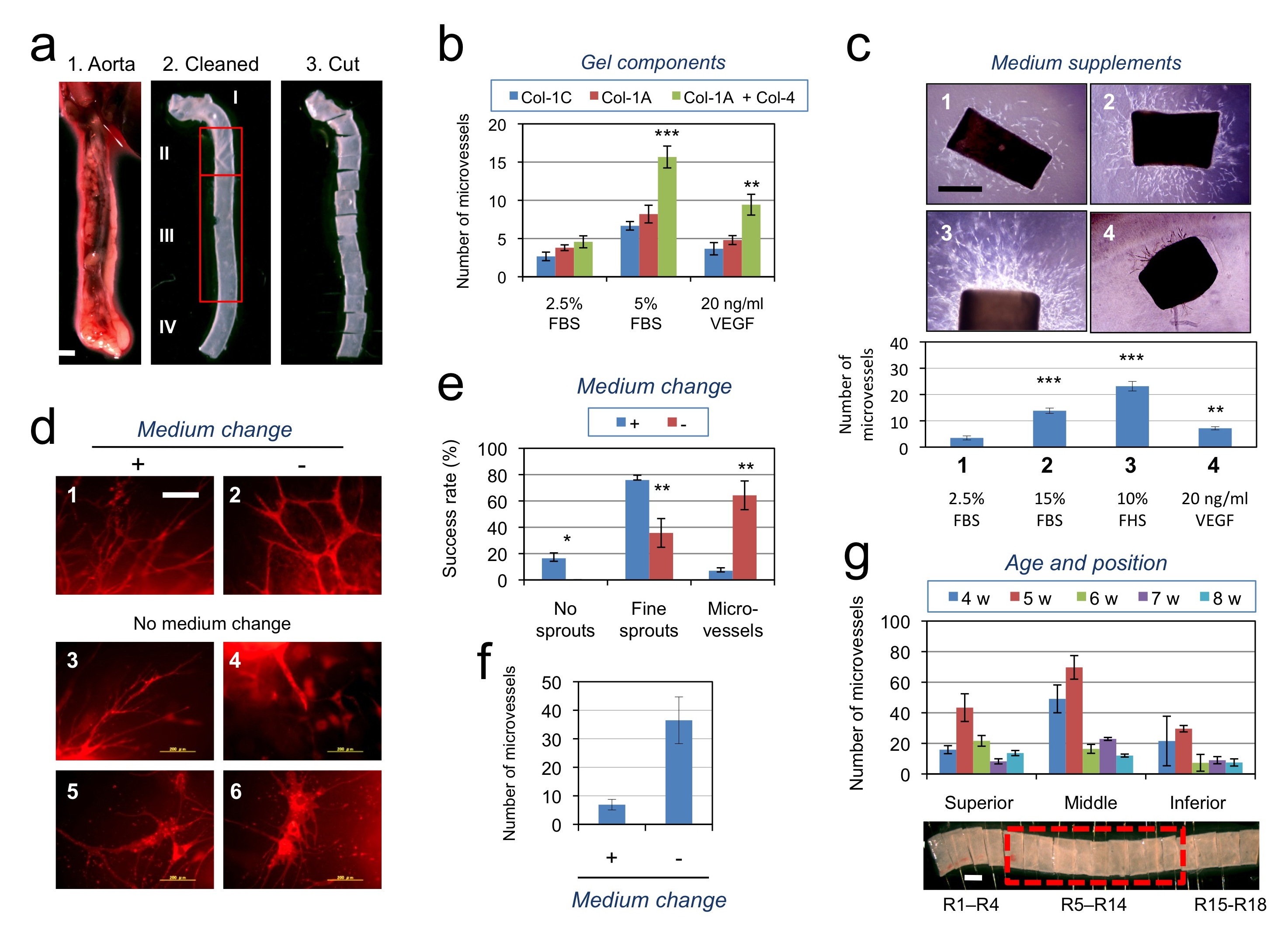
**

**Figure S2 | Optimizing ARA for ICR mice.** (a) Preparation of thoracic aorta from *Reck^CrER/+^;mTmG* ICR mice. Fresh aorta covered by adipose tissue (1) was cleaned taking care not to remove the adventitia tunica (2), cut into the rings of about 1-mm thick (3), and subjected to ARA. Four regions of aorta are indicated in panel 2: ascendant aorta (I), superior descendent aorta (II), middle descendent aorta (III), and inferior descendent aorta (IV). (b) Effects of gel composition and medium supplements. Freshly prepared aortic rings were embedded in semi-solid medium containing collagen of three different compositions (type 1C, type 1A, and type 1A plus 4), fed with medium supplemented with fetal bovine serum (FBS, two different concentrations) or vascular endothelial growth factor-A (VEGFa; R & D Systems), and incubated for 7 days with medium changes at days 5 and 10, followed by microvessel counting. Data represent more than four independent experiments with total of 65 rings prepared from 17 animals. (c) Effects of medium supplements. Freshly prepared aortic rings were embedded in the gel containing collagen type 1A and 4, fed with medium supplemented with FBS, fetal horse serum (FHS), or VEGFa at indicate concentration(s) and incubated in medium containing the same supplement with medium change at days 5 and 10. Phase-contrast images of typical rings (panels 1-4) and the number of microvessels at day 7 (bar graph, n=24 rings from 6 animals) are shown. (d) Effects of medium changes on microvessel morphology. *mT*-expressing aortic rings, pre-incubated overnight in serum-free medium, were subjected to ARA under the optimal gel components (collagens-1A and 4) and medium supplements (10% FHS); medium was either changed at days 5 and 10 (panel 1) or kept unchanged (panels 2-6). Micrographs were taken at day 7 focusing on morphology of sprouts (panels 1, 2) and various types of cells (panels 3-6). (e) The frequency of rings that gave rise to no sprouts, fine sprouts (as shown in d-1) or more robust protrusions (as shown in d-2, which we term “microvessels”). Data for the rings incubated with (+, n=12) or without (-, n=7) medium changes are compared. (f) Frequency of microvessels per ring from experiment e. (g) Effects of age of mice and position in aorta on microvessel number. Aortic rings from rostoral quarter (superior), caudal quarter (inferior), and the remaining area (middle) of aorta ( see the lower photograph of a sample at 8 weeks of age) obtained from mice at indicated age were subjected to ARA, and the number of microvessels were counted at day 7. n=3 aortas per group (total 243 rings). Scale bar: a, c, g 1 mm, d 200 µm. Data in bar graphs represent mean+SEM Data with significant difference as detected by Student’s t-Test are indicated as follows: * *P <* 0.05, ***P <* 0.01, *** *P <* 0.001.

**
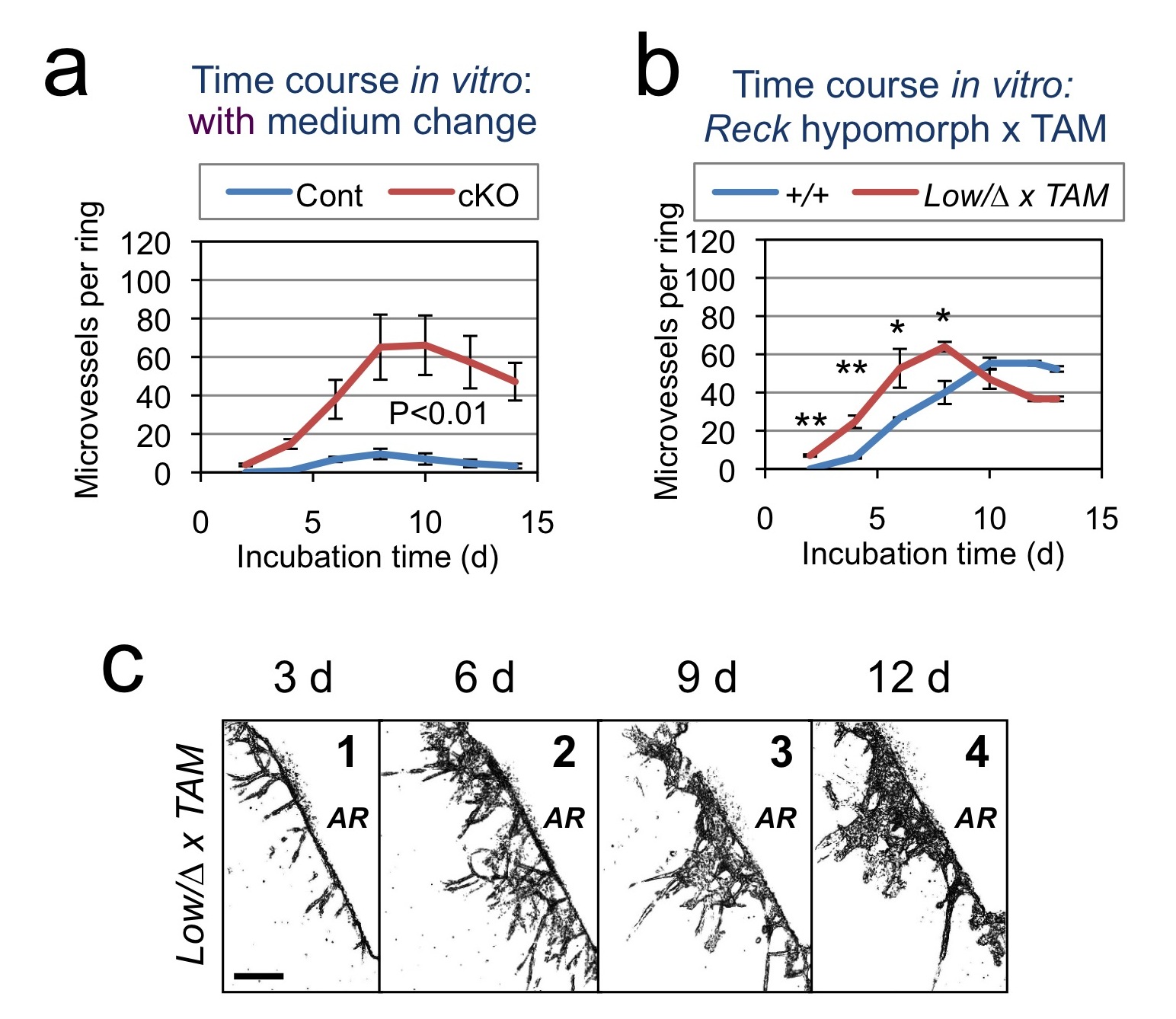
**

**Figure S3 | Effects of *Reck*-deficiency on microvessel formation in ARA.** (a) Time course of microvessel formation (number per ring) when medium was refreshed at days 5 and 10. Aortic rings from 5-weeks-old control (*Reck^+/CrER^;mTmG*, blue line, n=5) or cKO (*Reck^E1fx/CrER^;mTmG*, red line, n=3) mice were used. Note that cKO aortic rings could rapidly extend abundant microvessels under these conditions, while control aortic rings failed to do so. The reason for this difference has yet to be clarified. (b) Effects of *Reck*-deficiency on microvessel formation in ARA as examined using hypomorphic *Reck* mutant mice. Time course of microvessel formation (without medium changes) by aortic rings derived from the wild type (+/+) or *Reck^Low/∆^* (Low/∆) mice pretreated with tamoxifen. n=4. (c) Binary images of a typical region of a ring derived from tamoxifen-treated *Reck^Low/∆^* mice after indicated period of incubation in ARA (without medium changes). Phase-contrast images were converted to binary images using ImageJ software. Scale bar: 0.5 mm. In this mutant, basal *Reck* expression is about one forth of the wild type level, and tamoxifen induces acute inactivation of residual *Reck* expression. Note that the initial increase and subsequent decline in microvessel number seem to be accelerated (b**,** red line) as compared to the cKO samples (a, red line). Aggregation and thickening of microvessels also become even more pronounced (c).


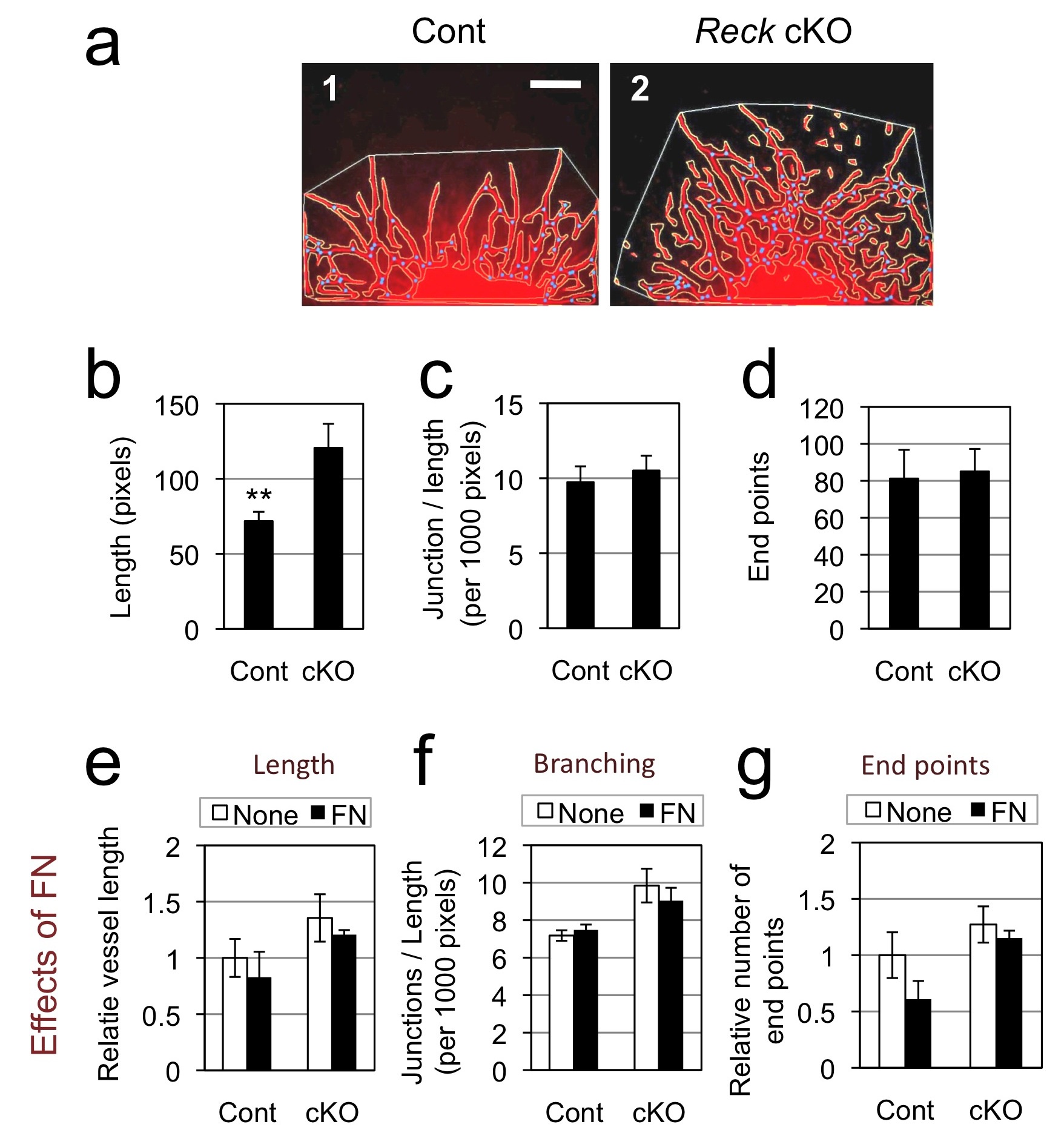


**Figure S4 | Morphometry of microvessels.** (a) Skeletonized images of a typical control (panel 1) and cKO (panel 2) ring after incubation for 10 days in ARA. Fluorescent micrographs were processed using AngioTool software. Scale bar: 1 mm. (b) Relative length of microvessels measured using ImageJ on low magnificantion images. Summary of 6 independent experiments (in total, n = 72 rings for Cont and 64 rings for cKO). **P<0.01. (c, d) Parameters measured using AngioTool. Junction/length (c) was calculated from the two parameters measured by AngioTool. Summary of 8 independent experiments (46 rings) for Cont and 7 experiments for cKO (43 rings). (e-g). Aortic rings from control (Cont) or *Reck*-deficient (cKO) mice were subjected to ARA without or with fibronectin (FN, 1 µg/ml), and indicated parameters were measured at day 10 as described above. Cont: n>14 rings (3 aortas); cKO: n>43 rings (6 aortas).


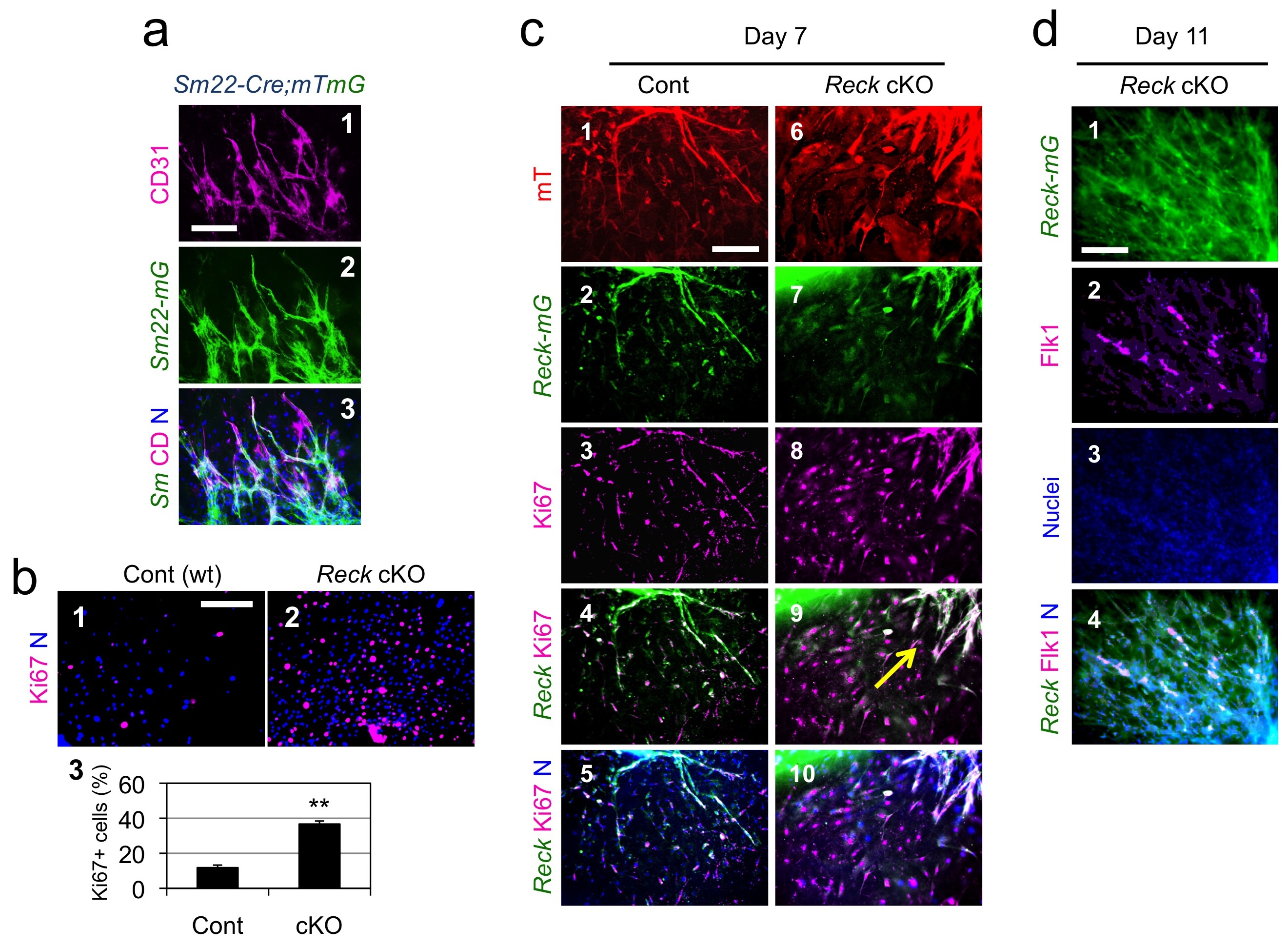


**Figure S5 | Immunofluorescent staining for CD31 and Ki67.** (a) Relationship between endothelial cells and mural cells in ARA. Aortic rings from 5-weeks old *mTmG;Sm22-Cre* mice were subjected to ARA and stained for CD31 (magenta) at day 11. Images of a typical sample are shown. Green signals (*Sm22-mG*) represent the cells of mural lineage. (b) Effects of *Reck* on cell proliferation in ARA. Wild type (1) and cKO (2) aortic rings cultured for 7 days were stained for a proliferation marker, Ki67 (magenta), followed by nuclear-counter-staining (blue). The proportion of Ki67-positive nuclei was estimated using ImageJ (3) and was found to be significantly increased in *Reck* cKO cells. **P=0.004. (c) Aortic rings from the control (*Reck^+/CrER^;mTmG*) or cKO (*Reck^E1fx/CrER^;mTmG*) mice were subjected to ARA and stained for Ki67 at day 7 (magenta in panels 3, 8). Note the substantial increase in proliferating *Reck-mG* (white) as well as proliferating non-*Reck-mG* (magenta) signals in the cKO samples (panels 9, 10). Arrow indicates prominent clusters of white and magenta signals associated with microvessels. (d) Flk1-positive cells in cKO microvessels. The cKO culture as used in Fig. 3b was immunostained for a vascular tip-cell marker, Flk1 (magenta). Flk1 signals are generally weak as compared to the control samples (as shown in Fig. 3c) and are not particularly abundant on the cells located at microvessel tips. Scale bar: a, b 100 µm, c 100 µm (Cont) 50 µm (cKO), d 50 µm.


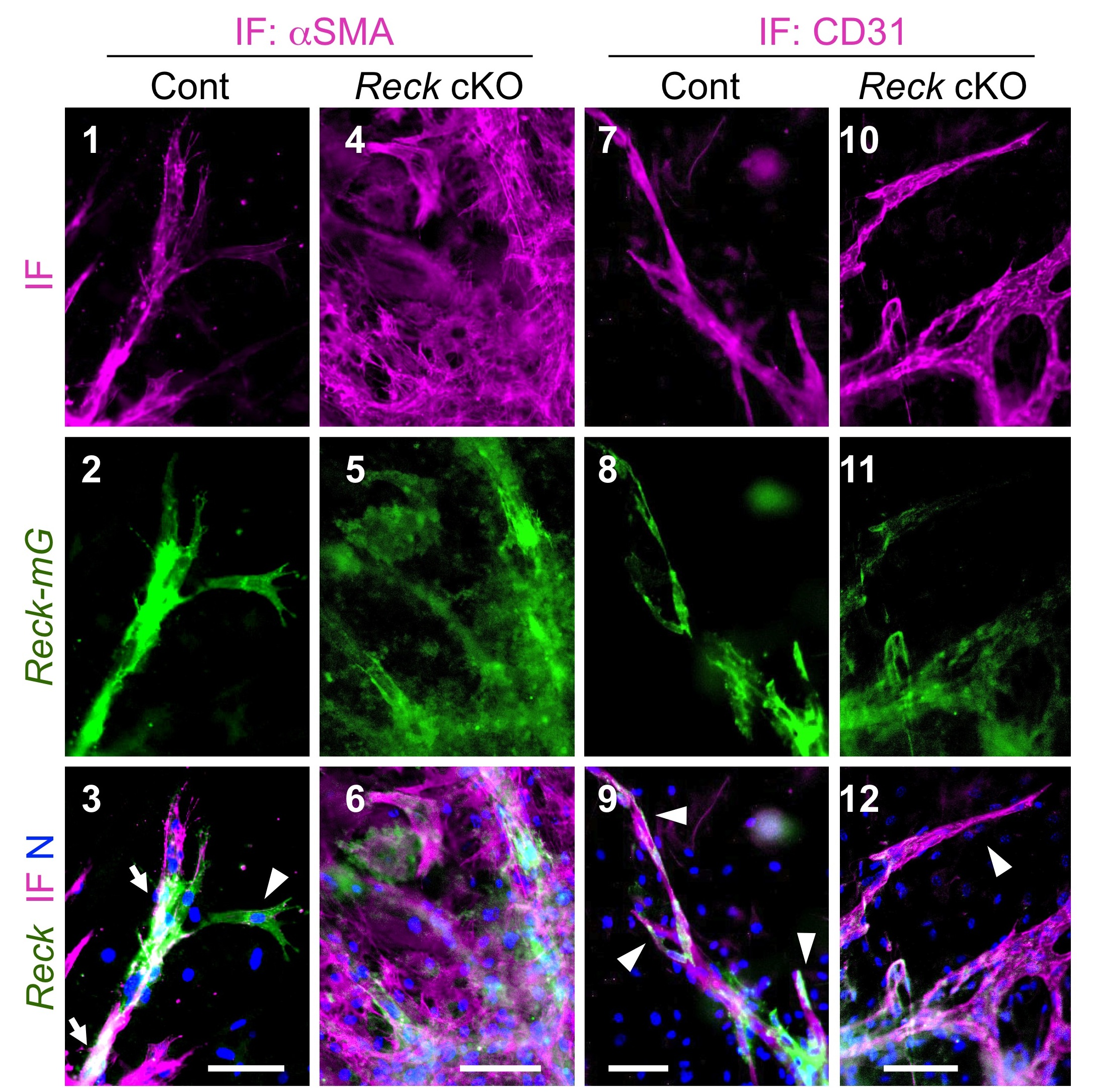


**Figure S6 | Expanded view of a section of each panel in Fig. 3b.** Aortic rings from the control (*Reck^+/CrER^;mTmG*) or *Reck* cKO (*Reck^E1fx/CrER^;mTmG*) mice were subjected to ARA and immunostained for αSMA (magenta in panels 1, 3, 4, 6) or CD31 (magenta in panels 7, 9, 10, 12) at day 11. Green signals represent *Reck-mG* cells*.* Scale bar: 50 µm. Arrows indicate Reck-mG/αSMA double positive cells surrounding the stalk. Arrowhead show cells near the tip of a sprout.


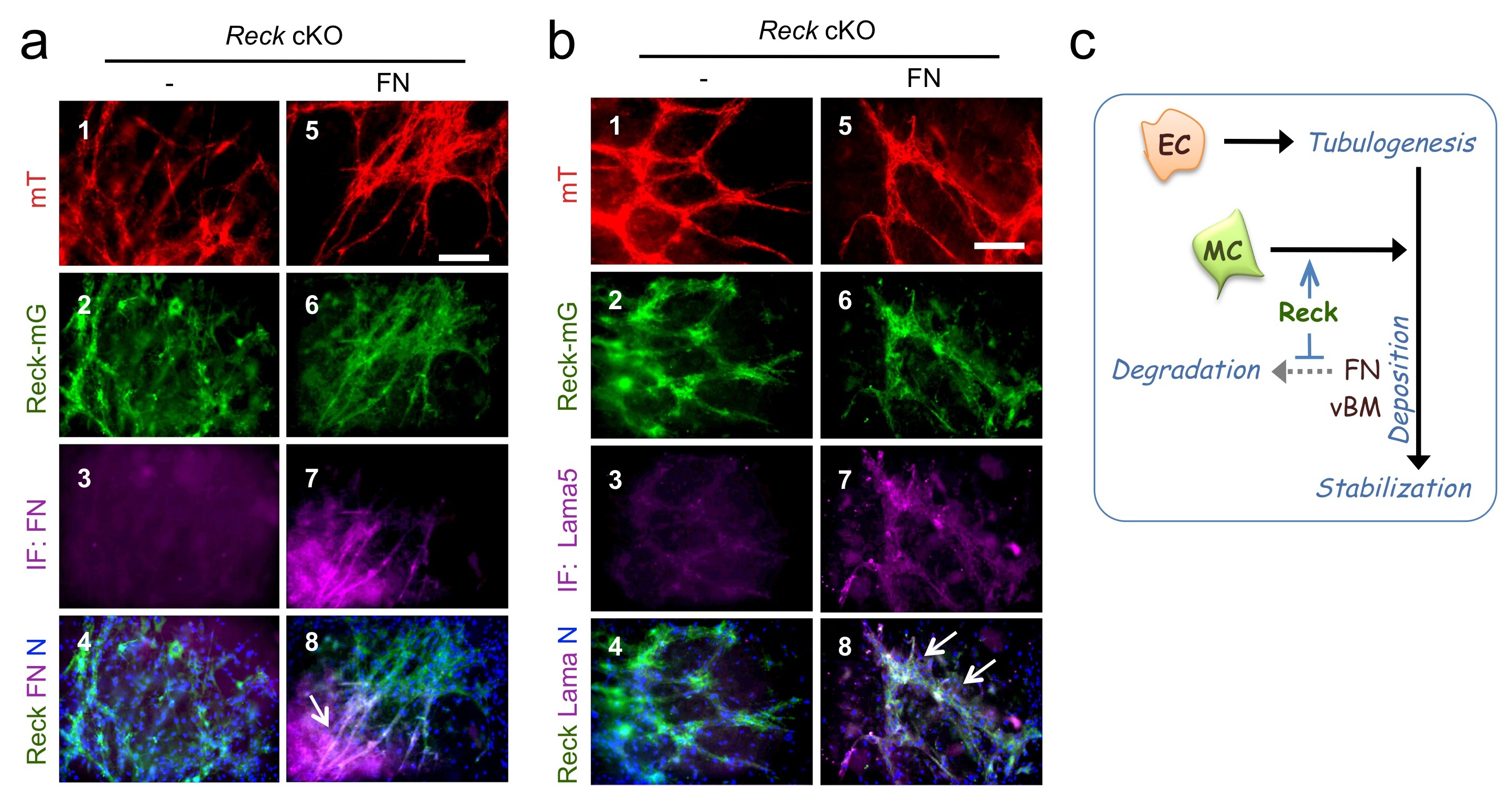


**Figure S7 | Effects of FN on the deposition of FN and Lama5 to cKO microvessels in ARA.** (a) Typical images of microvessels from cKO aortic rings after incubation for 13 days in the absence (1-4) or presence (5-8) of exogenous FN, followed by immunofluorescent staining for FN (magenta), and nuclear-counter-staining (blue). Top panels show *mT* cells representing the cells that had not experienced Cre-recombination, the second panels *Reck-mG* (green), and the bottom panels overlay of the *Reck-mG*, FN, and nuclear images. Note the white fibers that project into the magenta area (arrow in panel 8), indicating active FN-deposition on the microvessels associated with *Reck-mG* cells in the FN-rich gel. (b) Similar samples were stained for Lama5 deposition. Note that the white fibers in the overlaid images are conspicuous only when FN is present (arrows in panel 8), indicating that abundant FN is necessary and sufficient to promote Lama5-deposition (i.e., vBM formation) on cKO microvessels containing *Reck-mG* cells. Scale bar: A, B 50 µm. (c) A model to explain how *Reck* promotes vascular stabilization. Reck promotes (blue arrow) tight association of mural cells (MC) to the tubule-forming endothelial cells (EC), an event known to trigger perivascular FN-deposition^2,3^. Reck also protects FN from degradation, thereby promoting downstream events, including perivascular vBM-deposition, which helps establish vascular stability.


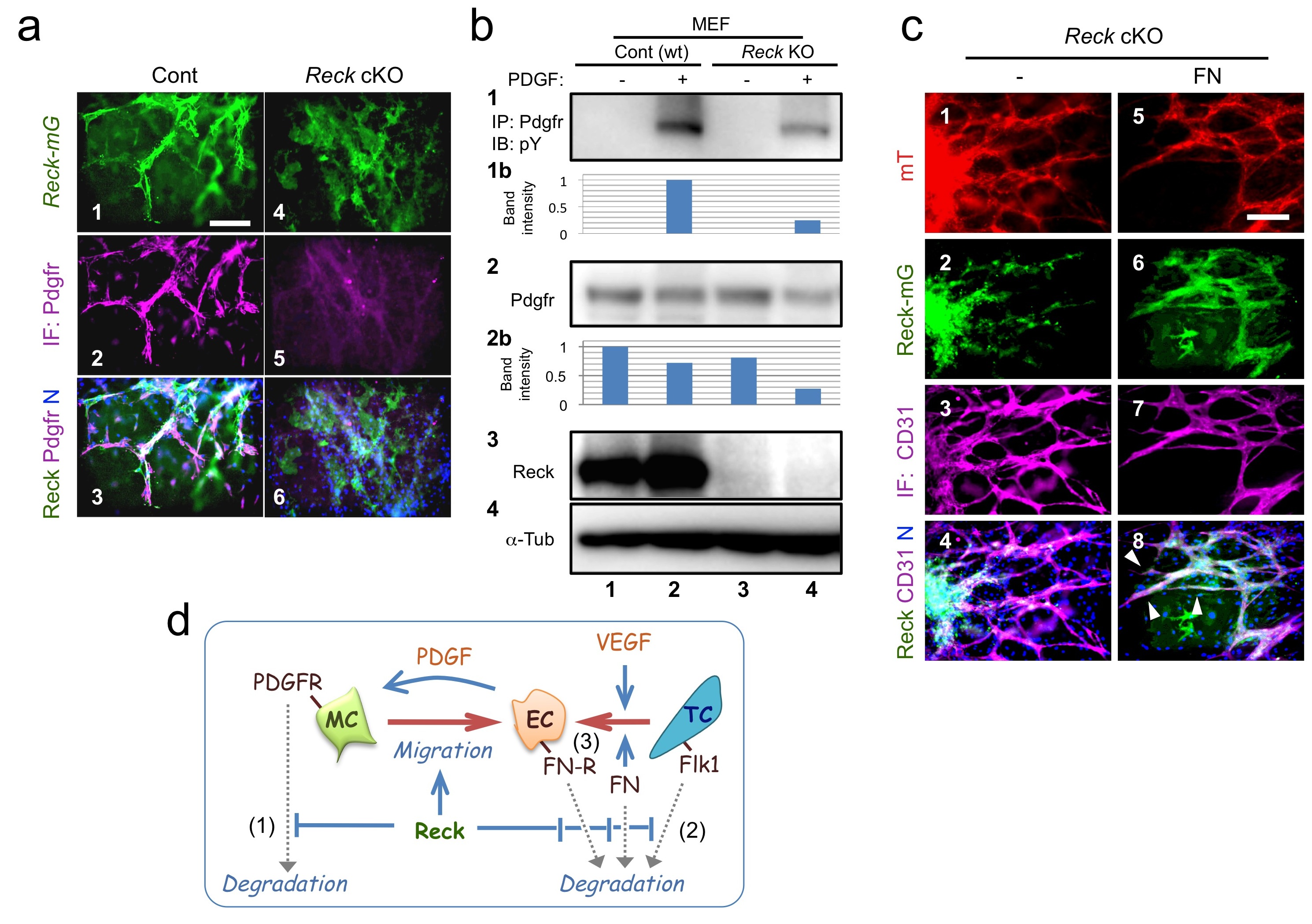


**Figure S8 | Possible mechanisms by which *Reck* promotes correct interactions between vascular cells.** (a) PDGF-receptor on *Reck-mG* cells. Control (1-3) and cKO (3-6) aortic rings were cultured as described in Fig. 3b for 14 days and stained for PDGF-receptor (magenta) followed by nuclear-counter-staining (blue). Note that in control culture, PDGF-receptor-signals are abundant on *Reck-mG* cells (green) associated with microvessels (white signals in panel 3), whilst dampened signals are found in cKO samples (6). (b) Effects of Reck on abundance and activation of PDGF-receptor in fibroblasts. Sub-confluent cultures of fibroblasts prepared from *wt* or *Reck*^-/-^ (KO) mice were serum-starved for 24 h and then incubated for 10 min in the same medium (-) or medium containing 10 µg/ml PDGF-BB (R&D Systems). Cell lysates were either immunoprecipitated with anti-PDGF-receptor followed by immunoblot assay using anti-phospho-tyrosine antibody (Cell Signaling Technology #9411; panel 1) or directly subjected to immunoblot assay using antibodies against either PDGF-receptor (2), Reck (3), or α-tubulin (4). Intensity of bands determined using ImageJ is presented in panels 1b (ratio of absolute values) and 2b (ratio of PDGFR value divided by α-tubulin value). Note the reduced signals in KO samples, especially in the presence of PDGF (lane 4 vs. 2). (c) Effects of FN on localization of endothelial *Reck-mG* cells. Typical images of microvessels from cKO aortic rings after incubation for 13 days in the absence (1-4) or presence (5-8) of exogenous FN, followed by immunofluorescent staining for CD31 (magenta), and nuclear-counter-staining (blue). Note that white signals (i.e., endothelial *Reck-mG* cells) could be found at or near microvessel tips (arrowheads) in samples incubated in FN-supplemented gel. Scale bar: a, b 50 µm. (d) Possible mechanisms by which Reck facilitates mural-endothelial and tip-stalk interactions. Reck protects PDGF-receptors on mural cells (MC), thereby keeping MC sensitive to the attractant (PDGF) produced by endothelial cells (EC). Reck also protects FN and Flk1 to stabilize the tip cell phenotype or to promote tip-stalk interaction directly^4^.

**References**

1 Yang, X., Gong, Y. & Friesel, R. Spry1 is expressed in hemangioblasts and negatively regulates primitive hematopoiesis and endothelial cell function. *PLoS One* 6, e18374 (2011).

2 Senger, D. R. & Davis, G. E. Angiogenesis. *Cold Spring Harb Perspect Biol* 3, a005090 (2011).

3 Stratman, A. N. & Davis, G. E. Endothelial cell-pericyte interactions stimulate basement membrane matrix assembly: influence on vascular tube remodeling, maturation, and stabilization. *Microsc Microanal* 18, 68-80 (2011).

4 Wijelath, E. S. *et al.* Novel vascular endothelial growth factor binding domains of fibronectin enhance vascular endothelial growth factor biological activity. *Circ Res* 91, 25-31 (2002).
